# ICEBERG study: an indirect adjusted comparison estimating the long-term benefit of esketamine nasal spray when compared with routine treatment of treatment resistant depression in general psychiatry

**DOI:** 10.3389/fpsyt.2023.1250980

**Published:** 2023-10-31

**Authors:** Albino J. Oliveira-Maia, Joachim Morrens, Benoit Rive, Yordan Godinov, Jedelyn Cabrieto, Nolen Perualila, Sebastien Barbreau, Siobhán Mulhern-Haughey

**Affiliations:** ^1^Champalimaud Research and Clinical Centre, Champalimaud Foundation, Lisbon, Portugal; ^2^NOVA Medical School, Faculdade de Ciências Médicas, NMS, FCM, Universidade Nova de Lisboa, Lisbon, Portugal; ^3^Janssen EMEA, Beerse, Belgium; ^4^Janssen EMEA, Paris, France; ^5^Janssen EMEA, Sofia, Bulgaria; ^6^Janssen EMEA, Dublin, Ireland

**Keywords:** treatment resistant depression, real-world evidence, indirect comparison, remission, response, esketamine nasal spray

## Abstract

**Background:**

Treatment resistant depression (TRD) affects 10–30% of patients with major depressive disorder. In 4-week trials, esketamine nasal spray (NS) was efficacious vs. placebo when both were initiated in addition to a new selective serotonin or serotonin norepinephrine reuptake inhibitor. However, comparison with an extended range of real-world treatments (RWT) is lacking.

**Methods:**

ICEBERG was an adjusted indirect treatment comparison using propensity score-based inverse probability weighting, performed on 6-month response and remission data from patients receiving esketamine NS plus oral antidepressant from the SUSTAIN-2 (NCT02497287; clinicaltrials.gov) study, compared with patients receiving other RWT from the European Observational TRD Cohort (EOTC; NCT03373253; clinicaltrials.gov) study. SUSTAIN-2 was a long-term open-label study of esketamine NS, while the EOTC was conducted at a time when esketamine NS was not available as RWT. Threshold and sensitivity analyses were conducted to assess how robust the primary analyses were.

**Results:**

Patients receiving esketamine NS had a higher probability of 6-month response (49.7% [95% confidence interval (CI) 45.6–53.9]) and remission (33.6% [95% CI 29.7–37.6]) vs. patients receiving RWT (26.4% [95% CI 21.5–31.4] and 18.2% [95% CI 13.9–22.5], respectively), according to rescaled average treatment effect among treated estimates. Resulting adjusted odds ratios (OR) and relative risk (RR) favoured esketamine NS over RWT for 6-month response (OR 2.756 [95% CI 2.034–3.733], *p* < 0.0001; RR 1.882 [95% CI 1.534–2.310], *p* < 0.0001) and remission (OR 2.276 [95% CI 1.621–3.196], *p* < 0.0001; RR 1.847 [95% CI 1.418–2.406], *p* < 0.0001). Threshold analyses suggested that differences between the two studies were robust, and results were consistent across extensive sensitivity analyses.

**Conclusion:**

ICEBERG supports that, at 6 months, esketamine NS has a substantial and significant benefit over RWT for patients with TRD. While results may be affected by unobserved confounding factors, threshold analyses suggested these were unlikely to impact the study conclusions.

To view an animated summary of this publication, please click on the Supplementary video.

## 1. Introduction

Approximately 10–30% of patients with major depressive disorder (MDD) develop treatment resistant depression (TRD), most frequently defined as a major depressive episode (MDE) failing to respond to two or more different antidepressants, given at adequate dose and duration (1–5). While multiple definitions of TRD exist, including biologic and clinical definitions (6, 7), this definition was supported by the Sequenced Treatment Alternatives to Relieve Depression (STAR*D) study, including 3,671 patients with a MDD for whom the probability of achieving response and remission fell from 48.6% and 36.8% with first-line therapy to 16.8% and 13.7% with third-line therapy, respectively (2). Indeed, several studies have shown that as the number of treatment failures increases, the likelihood of achieving response or remission decreases (2, 8, 9). Importantly, patients with TRD who experience treatment failure face a high clinical burden of disease, with higher rates of functional impairment and reduced health-related quality of life (3, 8, 10, 11). Furthermore, treatment failure is associated with a greater economic burden of disease, in part due to increased healthcare resource utilisation (3, 11, 12). It is therefore of great importance to identify the most efficacious treatments for patients with TRD, to increase the chance of patients achieving response or remission and thus reduce the burden of disease.

Currently, treatments prescribed for patients with TRD may include a wide variety of options, used in an individualised approach (13). Pharmacological treatments commonly include selective serotonin reuptake inhibitors (SSRIs), serotonin norepinephrine reuptake inhibitors (SNRIs), tricyclic antidepressants (TCAs) and antidepressants with other mechanisms of action (8, 13). Antidepressants can be prescribed as monotherapy or combined (13). Additionally, medications which were not developed initially as antidepressants but may have an antidepressant effect, namely antipsychotics and mood stabilisers, can be prescribed alongside an antidepressant as part of an augmentation therapy regimen (13).

Esketamine nasal spray (NS) is an N-methyl-D-aspartate (NMDA) receptor antagonist that targets the glutamatergic neurotransmitter system (14), rather than monoamines, which are the target for the most commonly used antidepressants (15). Esketamine NS, in combination with a SSRI or SNRI, obtained American and European-wide market approval specifically for TRD (14, 16, 17). Results from randomised controlled trials (RCTs) have demonstrated that in patients with TRD, esketamine NS has superior results compared with placebo when both are initiated in addition to a new SSRI/SNRI (18–21). However, most pivotal RCTs were short-term (4 weeks) and comparisons with other treatments are not yet available (19–22). As such, there is a gap in the literature regarding the evaluation of esketamine NS against current real-world treatments (RWT) during both acute and maintenance treatment phases.

The Indirect adjusted Comparison Estimating the long-term Benefit of Esketamine NS when compared with Routine treatment of TRD in General psychiatry (ICEBERG) aimed to address this evidence gap. ICEBERG analyses involved an adjusted indirect comparison of long-term (6-month) response and remission rates reported previously in a study of the efficacy of esketamine NS (plus oral antidepressant) (23), with equivalent data from a study assessing RWT for TRD (24). Equivalent, but distinct, analyses, in which polypharmacy (combination or augmentation) was used as a more specific RWT strategy comparator, are reported in a separate manuscript (25).

## 2. Methods

### 2.1. Study designs

An adjusted indirect treatment comparison (ITC) of esketamine NS with RWT strategies was performed using individual patient data from two studies of patients with TRD. SUSTAIN-2 (NCT02497287) was a long-term, open-label study of the safety and efficacy outcomes of esketamine NS in addition to a new antidepressant (SSRI or SNRI), which included patients in Europe, North and South America, Asia, Africa and Oceania (23, 26). The SUSTAIN-2 trial involved six-month follow-up of patients treated with esketamine NS, in line with prescribing information for TRD from the European Medicines Agency (EMA) and the Food and Drug Administration (FDA) (14, 17). In SUSTAIN-2, only patients who achieved treatment response at Week 4 were allowed to continue study treatment beyond this point in time (26), with no other pre-specified treatment continuation period, aligning with the esketamine NS EU label (14). For the ICEBERG analyses, even patients who did not reach a treatment response at Week 4 were included in the analysis using a non-responder imputation (NRI) approach. All direct-entry patients from SUSTAIN-2 who met the selection criteria at baseline were included in ICEBERG, while patients who entered SUSTAIN-2 from the TRANSFORM-3 phase 3 trial were excluded from analysis (Supplementary Figure 1A).

The European Observational TRD Cohort (EOTC) Study (NCT03373253) was a prospective, non-interventional, multicentre study in patients initiating a new, routine treatment for TRD in real-world clinical practice across several European countries (Supplementary Figure 1B) (8, 10, 24). The EOTC was conducted before the launch of esketamine NS in Europe, so no patient in the study received it.

The EOTC was selected for comparison with SUSTAIN-2 because the two studies were designed with similar inclusion and exclusion criteria and followed patients for at least 6 months. Both studies only included patients of 18 years of age or older, with a maximum age of 74 years for inclusion in the EOTC and no upper age limit for participation in SUSTAIN-2. Patients in both studies had a diagnosis of MDD without psychotic features, according to the Fifth Edition of the Diagnostic and Statistical Manual of Mental Disorders (DSM-5) or the Tenth Revision of the International Statistical Classification of Diseases and Related Health Problems (ICD-10) (27, 28). Furthermore, both studies used the same operational definition of TRD: failure to respond to antidepressant treatment, defined as a 25% or less improvement of depressive symptoms when treatment was working at its best, for at least two pharmacological treatments of sufficient duration (more than 6 weeks) and dosage in the current MDE, as documented in the Massachusetts General Hospital-Antidepressant Treatment Response Questionnaire (MGH-ATRQ). Patients in the EOTC were required to have a total Montgomery-Åsberg Depression Rating Scale (MADRS) score at baseline of ≥20, similar to the baseline score of ≥22 required for participation in SUSTAIN-2. Four patients (two from each study) who had only one treatment failure reported in the MGH-ATRQ were excluded from analyses. A summary table can be found in Supplementary Table 1, and full study designs and study flow diagrams can be seen in Supplementary Figures 1, 2, respectively.

### 2.2. Adjusted indirect treatment comparison

The adjusted ITC compared clinical outcomes from patients starting esketamine NS, plus an oral antidepressant, from SUSTAIN-2 with patients from the EOTC cohort starting an antidepressant treatment including at least one oral antidepressant medication. Patients from the EOTC who received only neurostimulation treatments, psychosocial interventions or an adjuvant treatment as monotherapy were excluded from the main analysis. This analysis compared 6-month data on response (≥50% improvement in total MADRS score compared to baseline) and remission (total MADRS score ≤ 10).

By default, remission and response outcomes were based on observed data at 6 months. However, for patients who dropped out for any reason before Month 6, response and remission data could not be collected (Table 1). In SUSTAIN-2, drop-outs marked the end of esketamine NS treatment, so a NRI approach was taken, considering these patients (including those who stopped treatment because of non-response at Week 4, as per protocol) as non-responders and non-remitters. Additionally, as per the study design, SUSTAIN-2 was terminated when at least 300 patients had received esketamine NS for 6 months, and at least 100 for 12 months. Patients who were ongoing at the time of study termination were reported as ‘study terminated by sponsor’. These patients were not considered for this study and only patients from SUSTAIN-2 enrolled more than 6 months before study termination were included in the present analysis.

**Table 1:** Data imputation and sensitivity analyses.

	Main analysis, SA1, SA2 and SA3	SA4	SA5	SA6
**Treatments prescribed at baseline in EOTC**	≥1 pharmacological treatment	≥1 pharmacological treatment	≥1 pharmacological treatment	All antidepressant treatments including neurostimulation and psychological therapies
**Data available at Month 6**				
**EOTC**	Observed data	Observed data	Observed data	Observed data
**SUSTAIN-2**	Observed data	Observed data	Observed data	Observed data
**Data missing due to patient drop-out before Month 6^a^**				
**EOTC**	Not included	Not included	Not included	Not included
**SUSTAIN-2**	NRI	NRI	NRI	NRI
**Data missing due to enrolment < 6 months before study termination^b^**				
**EOTC**	N/A	N/A	N/A	N/A
**SUSTAIN-2**	Not included	LOCF if discontinued due to study termination, NRI if discontinued for any other reason	Not included	Not included
**Data from patients who switched treatment or added another antidepressant or augmentation drug to original antidepressant**				
**EOTC**	Observed data	Observed data	NRI	Observed data
**SUSTAIN-2**	N/A	N/A	N/A	N/A

^a^In SUSTAIN-2, any patient stopping any part of their medication was dropped from the study. ^b^The end of SUSTAIN-2 occurred when ≥ 300 and ≥ 100 patients had received esketamine NS for 6 and 12 months, respectively. When this point was reached, all patients still in the study were withdrawn, with ‘study terminated by sponsor’ cited as reason for withdrawal. No patient selection was based on Week 4 evaluation, all patients from SUSTAIN-2 that met selection criteria were included. SUSTAIN-2 patients stopping for any other reason were imputed as NRI, even if included too close to study termination to ever reach the 6-month visit. EOTC, European Observational TRD Cohort; LOCF, last observation carried forward; NRI, non-responder imputation; NS, nasal spray; SA, sensitivity analysis; TRD, treatment resistant depression.

No such criterion was applied to data from the EOTC, since the study was terminated after the last enrolled patient reached the 6-month follow-up visit. To ensure a conservative approach in assessing the expected superiority of esketamine NS relative to RWT, drop-outs from the EOTC were considered non-informative and excluded from the main analysis, rather than using NRI. Finally, patients in the EOTC could switch treatment or add an antidepressant or augmentation drug to their original treatment and remain in the study. Considering the same conservative rationale, 6-month data collected from patients who experienced failure with initial treatment within the EOTC and changed treatment were nevertheless included in the main analysis, also reflecting the flexibility and adaptability inherent in RWT for TRD.

### 2.3. Covariates for adjustment

As patients were not randomised between the two studies, treatment cohorts were expected to be imbalanced. Cohorts may have differed at baseline on prognostic factors and could therefore be subject to confounding effects. To adjust for this, 17 covariates registered in both studies and covering patient characteristics including sociodemographics, clinical, psychometric, disease and treatment history, were used (Supplementary Table 2). Continuous covariates were coded in categories to accommodate any shape of association with tested outcomes and avoid assumptions regarding the nature of these associations. Only analyses from the final models, including all covariates, are presented here.

### 2.4. Main analyses

Two approaches were taken to adjust for imbalances between cohorts based on predicted prognostic factors. First, propensity score (PS)-based inverse probability weighting (IPW) was applied to balance the two cohorts on all available patient and disease characteristics at baseline. PS were first calculated based on multivariable logistic regression and were then transformed into weights using the 17 covariates to estimate the probability of receiving esketamine NS (SUSTAIN-2) or RWT (EOTC). PS distribution before and after reweighting can be found in Supplementary Figure 3. Treatment differences were estimated by reweighting observations in the EOTC according to IPW using PS, with SUSTAIN-2 as a reference. These remodelled EOTC data acted as a control arm for SUSTAIN-2, which corresponded to an average treatment effect among treated patients (ATT; i.e. patients in SUSTAIN-2) approach, with weights being rescaled to correspond to the original number of patients. Comparison outputs were expressed as probabilities for each treatment, with odds ratios (OR), relative risk (RR) and risk differences (RD), along with their respective 95% confidence intervals (CI). All outputs were estimated using weighted logistic regression. Number needed to treat (NNT) was derived from the RD and also reported. The ability of reweighting to reduce potential imbalances between studies was assessed by comparing the weighted distribution of PS of the reweighted populations and the standardised mean difference (SMD) on each covariate between the two studies, before and after reweighting.

When statistically significant differences were observed as favouring esketamine NS, threshold analyses were carried out. Simulations were performed in which the response rate or remission rate in the esketamine NS arm (SUSTAIN-2) was progressively decreased, while keeping the response or remission rate in the RWT arm (EOTC) unaltered. At each iterative rate decrease, the main analysis was replicated to check that statistical significance was maintained. This was performed separately for each efficacy indicator (OR, RR and RD). Differences between observed and simulated results were computed to understand how much lower response (or remission) rates in the esketamine NS arm could have been while still showing statistically significant superiority vs. RWT. Results from these threshold analyses were further illustrated by examining to what extent conclusions from the main analyses would be preserved in the presence of a hypothetical unobserved confounder that would be unbalanced between treatment arms and have an impact on main outcomes.

### 2.5. Multivariable analysis

The second approach used a multivariable logistic regression model, including the same 17 covariates, and using pooled individual patient data from the two studies to compare esketamine NS with RWT. This allowed estimation of the adjusted OR to quantify the relative treatment effect, accounting for imbalances between both cohorts. These multivariable models additionally provided estimates of the association of each of the covariates with the outcomes of interest.

### 2.6. Sensitivity analyses

Six sensitivity analyses (SA) were performed using various PS reweighting methods and alternative data handling approaches, to assess the robustness of the conclusions from the main analysis (Table 1). IPW methods were used to produce different estimates of treatment effect in different pseudo-populations, namely rescaled average treatment effect among control (ATC; SA1), stabilised average treatment effect (sATE; SA2), and average treatment effect among the overlap population (ATO; SA3).

In the fourth SA (SA4) patients who were enrolled in SUSTAIN-2 less than 6 months before study termination were re-included in the analysis, using a last observation carried forward (LOCF) approach if they withdrew due to study termination, and considered as a non-responder if they withdrew for any other reason. In SA5, patients in the EOTC who switched from, combined or augmented their initial baseline treatment were considered as treatment failures and handled using an NRI approach. Data from EOTC dropouts were treated in the same manner as for all other analyses and were excluded in this SA. In SA6, data from patients who received only neurostimulation treatments, psychosocial interventions, or an antipsychotic as monotherapy in the EOTC were also included in the analyses, using the same data handling convention as for the main analysis.

## 3. Results

### 3.1. Baseline characteristics and performance of reweighting

**Figure 1:**
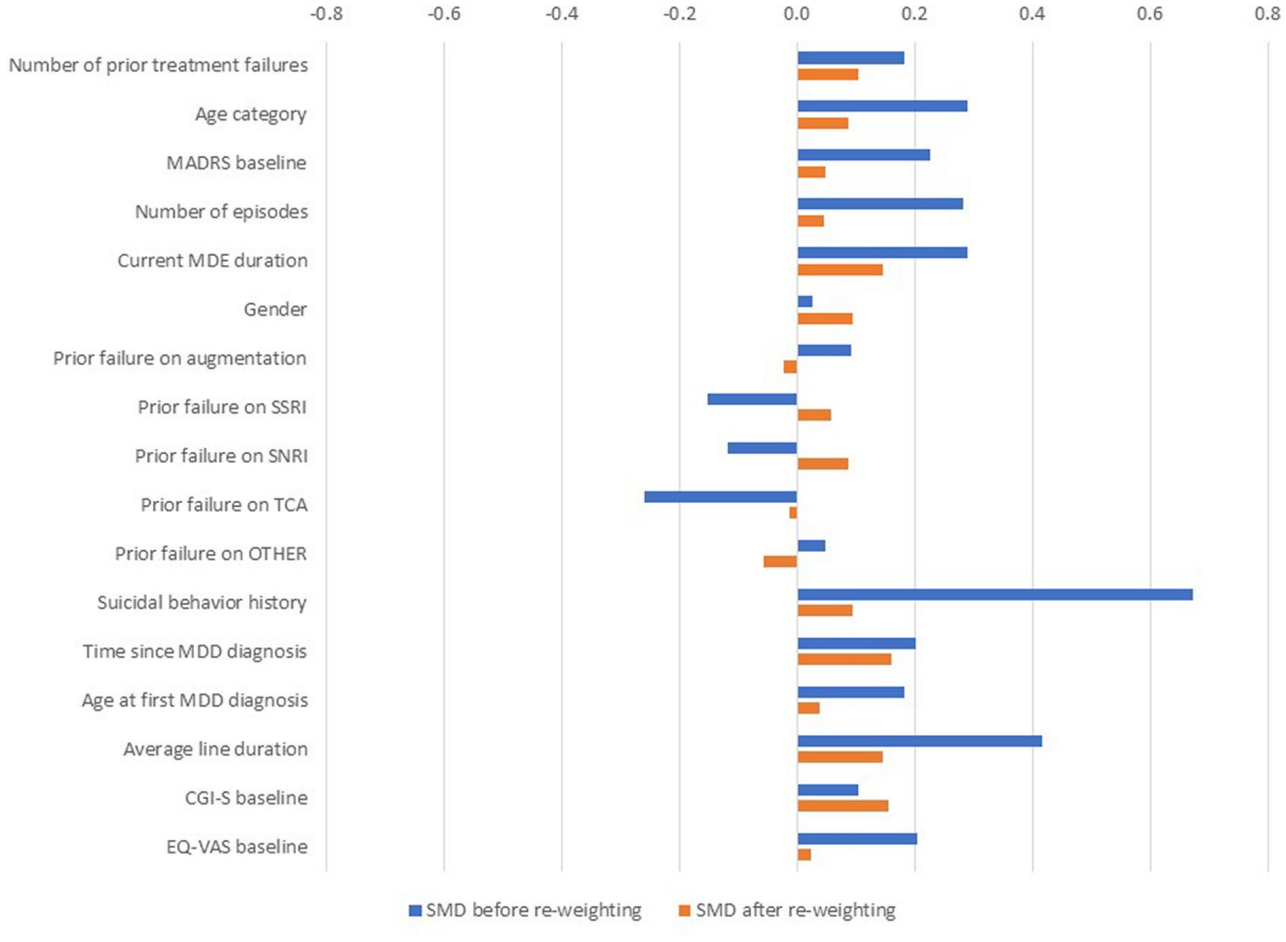
Standardised mean differences for observed and ATT-weighted cohorts. ^a^Prior failure on ‘other’ AD included trazodone, nefazodone, vilazodone, bupropion, mirtazapine, mianserin, opipramol, agomelatine, tianeptine, reboxetine and vortioxetine. The central vertical line represents esketamine NS data; all other data points represent data from patients prescribed RWT, relative to esketamine NS. AD, antidepressant; ATT, rescaled average treatment effect among treated; CGI-S, Clinical Global Impression-Severity; EOTC, European Observational TRD Cohort; EQ-VAS, EuroQol Visual Analogue Scale; MADRS, Montgomery-Åsberg Depression Rating Scale; MDD, major depressive disorder; MDE, major depressive episode; NS, nasal spray; RWT, real world treatment; SMD, standardised mean differences; SNRI, serotonin-norepinephrine reuptake inhibitor; SSRI, selective serotonin reuptake inhibitor; TCA, tricyclic antidepressant; TRD, treatment resistant depression.

Study flow diagrams for patients included in ICEBERG from either study can be found in Supplementary Figure 2. All patient demographics, clinical features of patients’ current MDE and history of treatment for MDD were similar between studies at baseline (Table 2). Furthermore, the 17 baseline covariates could be included in the PS estimation, and the ATT PS reweighting aligned the distribution of the EOTC population covariates more closely with that of the SUSTAIN-2 population (Figure 1). Indeed, after reweighting, all SMD were between −0.2 and + 0.2, indicating that none of these differences would be clinically detectable (29, 30). Similar results were seen with other IPW methods (sATE, ATC and ATO; data not shown). The PS distribution for the two populations is shown in Supplementary Figure 3, where a larger overlap in distributions after reweighting can be observed. Similar results were observed for SA1, SA2 and SA3 (data not shown).

**Table 2:** Baseline patient characteristics.

Category	RWT^a^	RWT	Esketamine NS	Esketamine NS
Mean (SD), unless otherwise stated	Main analysis, sensitivity analysis 1, 2, 3, 4 and 5	Sensitivity analysis 6	Main analysis, sensitivity analysis 1, 2, 3, 5 and 6	Sensitivity analysis 4
	N = 307	N = 336	N = 559	N = 689
**Age, years**	51.1 (10.5)	50.9 (10.7)	49.8 (12.7)	49.4 (12.6)
**Gender, %** **(*n*)**				
Female	62.2 (191)	61.3 (206)	63.5 (355)	63.4 (437)
**Age at diagnosis of MDD, years**	37.7 (13.1)	37.6 (12.9)	35.0 (13.4)	34.7 (13.1)
**Time since first diagnosis of MDD, years**	13.5 (10.9)	13.2 (10.8)	14.7 (11.4)	14.7 (11.4)
**Total MADRS score**	32.0 (5.9)	32.0 (5.9)	31.2 (5.0)	31.2 (5.0)
**Total number of failures in current MDE**	2.7 (0.9)	2.7 (0.9)	2.6 (1.0)	2.7 (1.1)
**CGI-S score**	4.8 (0.7)^b^	4.8 (0.7)^b^	4.8 (0.7)	4.9 (0.7)
**EQ-VAS score**	41.2 (18.8)^c^	41.7 (19.1)^c^	44.4 (19.8)	44.0 (19.8)
**Total number of MDE**	4.1 (4.3)^d^	4.1 (4.5)^d^	4.1 (3.3)^d^	4.1 (3.6)^d^
**Duration of current MDE, years**	2.7 (4.0)	2.8 (4.2)	2.5 (4.2)	2.8 (4.6)
**History of suicidality (based on C-SSRS; lifetime), %** **(*n*)**				
No event	44.3 (136)^e^	42.0 (141)^f^	61.2 (342)	59.9 (413)
Suicidal ideation	30.6 (94)^e^	31.3 (105)^f^	23.4 (131)	24.4 (168)
Suicidal behaviour	7.8 (24)^e^	9.2 (31)^f^	15.4 (86)	15.7 (108)
**Average duration of each treatment line during current MDE,^g^ weeks**	51.5 (68.0)	54.4 (77.8)	43.2 (68.6)	47.4 (73.7)
**Prior failure on augmentation drug, %** **(*n*)**	12.7 (39)	12.8 (43)	15.9 (89)	17.7 (122)
**Prior failure on SSRI, %** **(*n*)**	81.4 (250)	81.0 (272)	75.1 (420)	75.6 (521)
**Prior failure on SNRI, %** **(*n*)**	56.0 (172)	56.5 (190)	50.1 (280)	50.9 (351)
**Prior failure on TCA, %** **(*n*)**	16.3 (50)	15.8 (53)	7.9 (44)	8.1 (56)
**Prior failure on other treatments^h^,** **%** **(*n*)**	49.5 (152)	50.3 (169)	51.9 (290)	51.5 (355)

Baseline patient characteristics in this table are before adjustment. ^a^In SA5, six additional patients who received RWT were included in the analysis as they switched treatment within 6 months but did not have a Month 6 MADRS score recorded and were therefore recorded as non-responders using NRI. These data had minimal impact on overall patient baseline characteristics. ^b^CGI-S data were missing for two patients in the EOTC. ^c^EQ-VAS score data were missing for five patients in the EOTC. ^d^Total number of MDE data were missing for four patients in the EOTC and one patient in SUSTAIN-2. ^e^C-SSRS data were missing for 53 patients in the EOTC. ^f^C-SSRS data were missing for 59 patients in the EOTC for SA6. ^g^Every patient received multiple treatment lines during their current MDE, so these data represent the average duration of each individual treatment line and not the overall duration of current MDE. ^h^Other treatments included trazodone, nefazodone, vilazodone, bupropion, mirtazapine, mianserin, opipramol, agomelatine, tianeptine, reboxetine and vortioxetine. CGI-S, Clinical Global Impression-Severity; C-SSRS, Columbia-Suicide Severity Rating Scale; EOTC, European Observational TRD Cohort; EQ-VAS, EuroQoL-visual analogue scale; MADRS, Montgomery-Åsberg Depression Rating Scale; MDD, major depressive disorder; MDE, major depressive episode; NRI, non-responder imputation; NS, nasal spray; RWT, real-world treatment; SA, sensitivity analysis; SD, standard deviation; SNRI, serotonin-norepinephrine reuptake inhibitor; SSRI, selective serotonin reuptake inhibitor; TCA, tricyclic antidepressant; TRD, treatment resistant depression.

### 3.2. Probabilities of response and remission (IPW and ATT)

**Figure 2:**
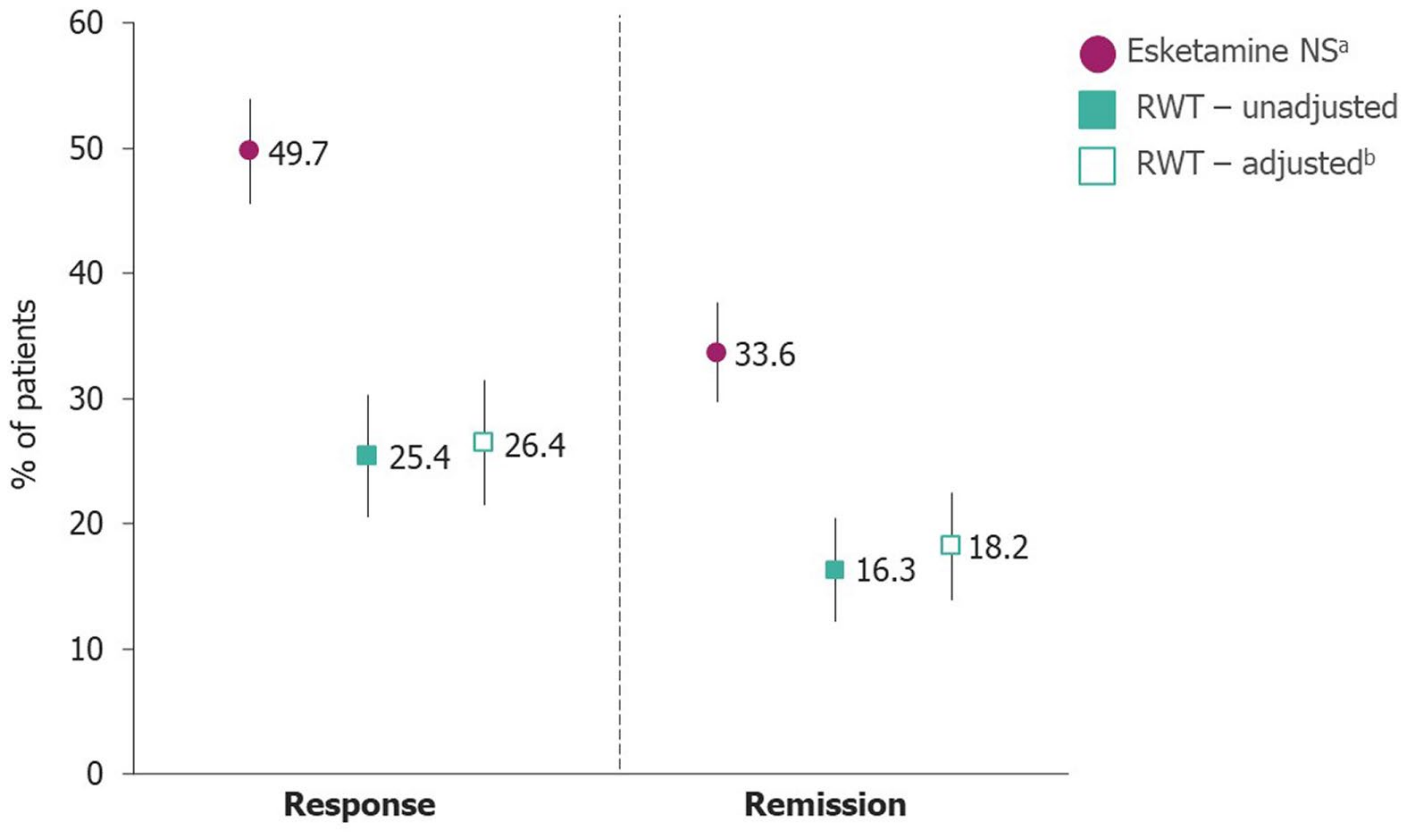
Probability of response and remission. Error bars represent upper and lower 95% CIs. Missing data were handled as per Table 1. ^a^Given in combination with an SSRI or SNRI. ^b^RWT data were adjusted using the ATT covariate adjustment method. ATT, rescaled average treatment effect among treated; CI, confidence interval; NS, nasal spray; RWT, real-world treatment; SNRI, serotonin norepinephrine reuptake inhibitor; SSRI, selective serotonin reuptake inhibitor.

In the main analysis, 278/559 (49.7%) patients receiving esketamine NS experienced response at Month 6 compared with 78/307 (25.4%) of patients receiving RWT. Following reweighting (IPW ATT), the estimated probability of response for patients receiving RWT was 26.4% (95% CI 21.5–31.4; Figure 2). Statistical comparison between the two revealed patients receiving esketamine NS had significantly better outcomes (*p* < 0.0001) compared with RWT in terms of 6-month response (Table 3). Indeed, there was a statistically significant benefit in response for esketamine NS over RWT, with a RR of 1.882 (95% CI 1.534–2.310) and a NNT of 5 (95% CI 4–6).

**Table 3:** Chance of response and remission at Month 6.

Esketamine NS^a^ vs. RWT	Response	Remission
**OR (95% CI); *p* value**	2.756 (2.034–3.733); < 0.0001	2.276 (1.621–3.196); < 0.0001
**RR (95% CI); *p* value**	1.882 (1.534–2.310); < 0.0001	1.847 (1.418–2.406); < 0.0001
**RD (95% CI); *p* value**	0.233 (0.169–0.298); < 0.0001	0.154 (0.096–0.213); < 0.0001
**NNT (95% CI)**	5 (4–6)	7 (5–11)

Missing data were handled as per Table 1. RWT data were adjusted using the ATT covariate adjustment method. ^a^Given in combination with an SSRI or SNRI. OR > 1, RR > 1 and RD > 0 all indicate esketamine is superior to the comparator treatment. ATT, rescaled average treatment effect among treated; CI, confidence interval; NNT, number needed to treat; NS, nasal spray; OR, odds ratio; RD, risk difference; RR, relative risk; RWT, real-world treatment; SNRI, serotonin-norepinephrine reuptake inhibitor; SSRI, selective serotonin reuptake inhibitor.

Remission rate at Month 6 was 33.6% (188/559) for patients receiving esketamine NS compared with 16.3% (50/307) for RWT before reweighting, and 18.2% (95% CI 13.9–22.5) after reweighting (IPW ATT; Figure 2). This resulted in a statistically significant benefit for esketamine NS over RWT (*p* < 0.0001; Table 3) in terms of 6-month remission, with a RR of 1.847 (95% CI 1.418–2.406) and NNT of 7 (95% CI 5–11).

Results based on alternative IPW algorithms (SAs 1–3) and across SAs 4–6 were consistent with the main analysis (Supplementary Tables 3–5).

As esketamine NS displayed statistically significant superiority over RWT for both remission and response, threshold analyses were conducted for both outcomes. Differences between observed probabilities for esketamine NS and lowest significant simulated probabilities that were still significantly higher than RWT ranged from 9.5 and 9.7% for remission, and 16.6 and 17.0% for response (Supplementary Table 6).

### 3.3. Multivariable analysis of response and remission

**Figure 3:**
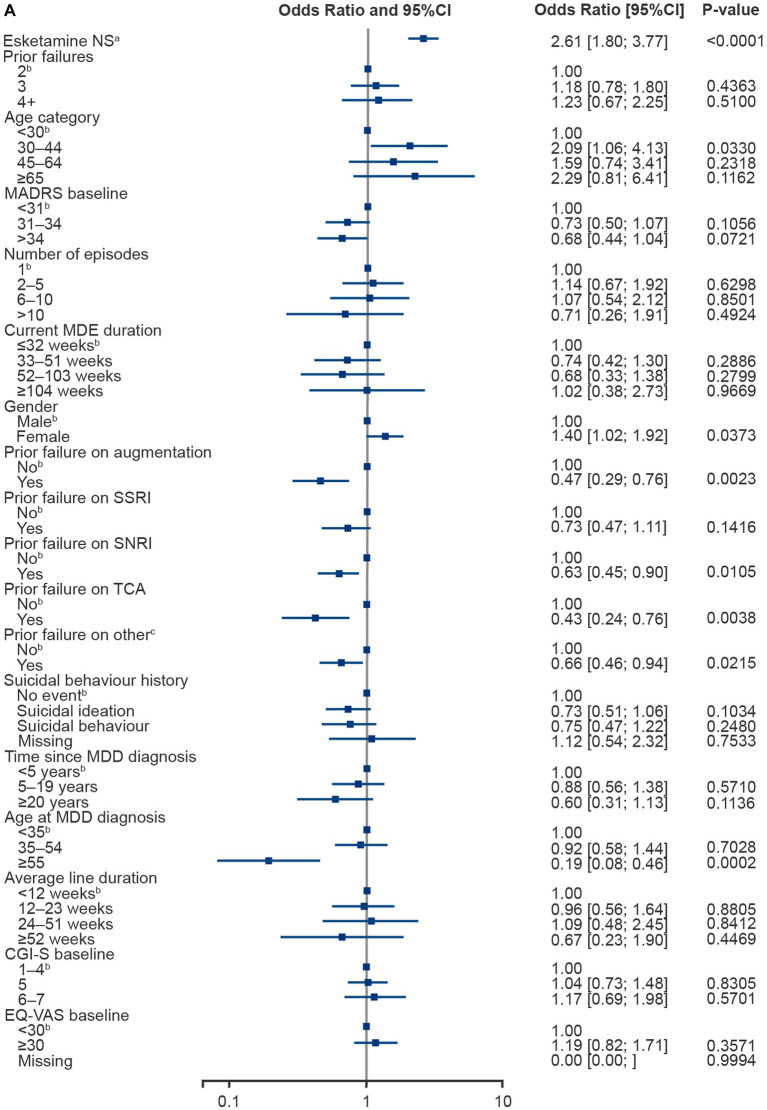

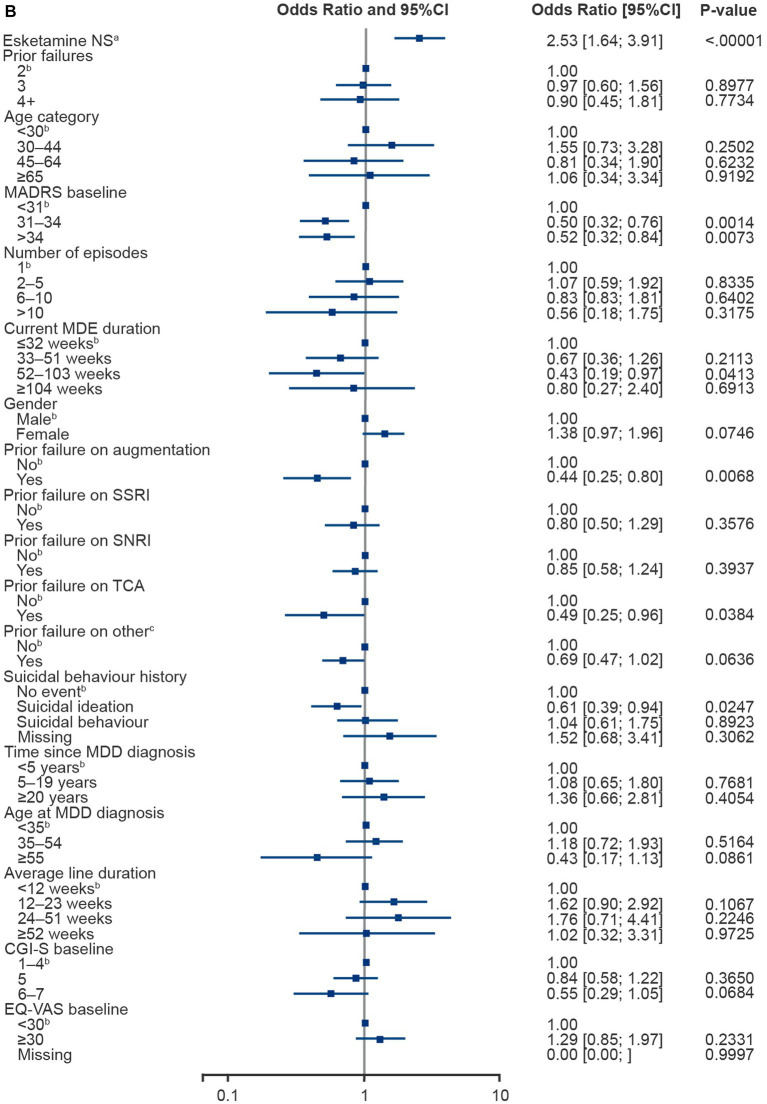
Multivariable logistic regression plots for 6-month response **(A)** and remission **(B)**. RWT excludes esketamine NS. ^a^Given in combination with an SSRI or SNRI. ^b^Reference value. ^c^Prior failure on ‘other’ included trazodone, nefazodone, vilazodone, bupropion, mirtazapine, mianserin, opipramol, agomelatine, tianeptine, reboxetine and vortioxetine. CGI-S, Clinical Global Impression-Severity; CI, confidence interval; EQ-VAS, EuroQol Visual Analogue Scale; MADRS, Montgomery-Åsberg Depression Rating Scale; MDD, major depressive disorder; MDE, major depressive episode; NS, nasal spray; RWT, real world treatment; SNRI, serotonin-norepinephrine reuptake inhibitor; SSRI, selective serotonin reuptake inhibitor; TCA, tricyclic antidepressant.

Variables were included in the analysis sequentially by rank, and all were included in the analyses. The ORs in the multivariable logistic regression models were significantly in favour of esketamine NS (Figure 3), with an OR of 2.61 (95% CI 1.80–3.77, *p* < 0.0001) for 6-month response and 2.53 (95% CI 1.64–3.91, *p* < 0.0001) for remission. Esketamine NS was the largest predictor of response and remission.

Age at MDD diagnosis (≥55 years), male gender and previous treatment failures with augmentation, SNRIs, TCAs or other antidepressants were all significantly associated with a lower chance of achieving response. Higher baseline MADRS score (≥31) and previous treatment failures with augmentation or TCAs were significantly associated with a lower chance of achieving remission. Patients with an MDE lasting 52 to 103 weeks had significantly lower chances of achieving remission compared to those with an MDE lasting <32 weeks. Prior history of suicidal ideation, but not suicidal behaviour, at baseline was also associated with a lower chance of experiencing remission (Figure 3).

## 4. Discussion

### 4.1. Summary of main findings

In this ITC, esketamine NS demonstrated substantial and significant benefit over RWT for patients with TRD in achieving both response and remission. The probabilities of response and remission for patients receiving esketamine NS were higher than the adjusted estimated probabilities (ATT estimates) for patients receiving RWT, with all efficacy indicators (OR, RR and RD) indicating a statistically significant and clinically important benefit of esketamine NS over RWT for both outcomes. The significant association of positive long-term outcomes with esketamine NS treatment was further supported in multivariable logistic regression analysis by favourable adjusted ORs for response and remission.

### 4.2. Summary of other findings

Multivariable logistic regression of pooled data from the two studies identified other risk factors associated with response or remission. Patients over 55 years of age at first MDD diagnosis were more likely to experience treatment failure at Month 6. The reasons for this are unclear, but potentially include age-related brain changes or higher rates of comorbidity and concomitant medications among older patients, leading to limited treatment options (31, 32). Additionally, prior failure of TCAs or augmentation was associated with a lower likelihood of response and remission on a new treatment. The number of prior treatment failures did not significantly influence the chance of 6-month response or remission in the full model. However, when this variable was considered in isolation from prior treatment failures of the distinct medication groups, there was a significant effect of prior failures on response and remission (data not shown).

### 4.3. Propensity score-based IPW methodology

ITCs are robust and proven methods of comparing treatment effects from different data sets, are supported by the NICE guidelines and are a part of the recommendations from several Health Technology Assessment (HTA) bodies for generating valid comparative data (33–38). ITCs used in HTA assessments most frequently use the approach of anchored methodology, comparing relative treatment effects between randomised trials vs. a common comparator, based on the aggregated results from both trials. The adjusted ITC methodology used in ICEBERG differed in that it used data from an unanchored comparison (the trials did not have a common comparator) of individual patient level data. Indirect comparative analyses are often confounded by differences in the patient populations under comparison. However, both studies included here used similar inclusion and exclusion criteria. Nevertheless, since access to the individual patient level data was available for both studies, adjustments of potential confounders was possible. Models using the classic covariate adjustment approach may be overfitted when the number of covariates is large compared with the number of patients or outcome events (39). To overcome this potential problem, PS methods were used which aimed to estimate the true treatment effect (39). Patient characteristics were ranked in advance of analysis by clinical opinion to ensure the most relevant were included in the model first in the case where not all variables could be included. This approach proved unnecessary as the final PS models were able to accommodate all 17 covariates to provide thorough readjustment of patient data into pseudo-populations. As the two observed study populations were well matched at baseline, minimal changes in comparisons were observed following PS reweighting, supporting the robustness of our analyses.

PS methods can only include observed patient characteristics. Unmeasured variables may still have acted as confounders but were not accounted for. Threshold analyses were thus carried out to provide perspective on the potential impact of unobserved confounders. The threshold analysis can be interpreted in two ways. First, if 9.5% fewer patients were in remission or 16.6% fewer showed response in SUSTAIN-2, esketamine NS would still have a statistically significant benefit over RWT. Second, based on these analyses, any unmeasured characteristics would need an extremely large effect size to negate the significant degree of benefit observed for esketamine NS over RWT. Indeed, if a potential unobserved confounder existed that was 30% more prevalent in SUSTAIN-2 than in EOTC, and that would increase the chance of remission by 30%, it would create an artificial overestimation of the remission rate for esketamine NS in SUSTAIN-2 of +9% (30% × 30%). This would still be less than the difference between observed remission rate for esketamine NS and the lowest simulated remission rate of esketamine NS, and still significant over RWT (ranging between 9.5 and 9.7%). Thus, it would still not change the conclusion of statistically significant superiority of esketamine NS over RWT. For response, differences between observed rates and lowest still significant simulated rate ranged between 16.6 and 17.0%. Thus, even a hypothetical unobserved confounder that would be up to 40% more prevalent among esketamine NS patients and increased chances of response by 40%, while artificially inflating response rate for esketamine NS by 16% (40% × 40%), would not change the conclusions of the analysis.

SAs using alternative PS-based reweighting algorithms were implemented (SAs 1–3), as well as three further SAs (SAs 4–6) with less conservative approaches to data handling and patient selection. The main analysis was determined to be the most appropriate approach, as it was the most conservative methodology that involved the fewest assumptions. No SA changed the conclusion that esketamine NS was superior to RWT, further supporting the robustness of the findings from the main analysis.

### 4.4. Strengths and limitations

Limitations must be considered to interpret these findings. In addition to the use of esketamine NS, several other factors may have contributed to higher rates of responses and remission in SUSTAIN-2. Patients in the SUSTAIN-2 clinical trial setting may have shown increased compliance and motivation to continue their initial prescribed treatment, due to both the nature of clinical trial management and the high frequency of interaction with a healthcare professional (40). However it must be considered that increased visits would also be expected for esketamine NS treatment in a real-world setting, where it should be administered under the direct supervision of a healthcare professional (14, 17). In any case, the threshold analyses presented here reveal that these findings were robust; even if there were other factors contributing to the results (e.g. being in a trial environment), the results would most likely remain significant if these factors were not present.

Furthermore, it must be considered that quite conservative options regarding data analyses were taken. Indeed, as per study design, in SUSTAIN-2 only patients who achieve response based on MADRS at Week 4 were allowed to continue beyond the esketamine NS treatment phase into the maintenance phase (14). For the ICEBERG analyses, all patients from SUSTAIN-2 that met the selection criteria were included, with drop-outs, including Week 4 non-responders, being considered non-responders at Month 6. On the other hand, patients enrolled in the EOTC could change treatment multiple times and were still considered responders if response was achieved by Month 6. Furthermore, drop-outs were not considered for analyses, rather than using the non-responder imputation approach used for SUSTAIN-2 data. Our approach was therefore conservative in terms of assessing superiority for esketamine NS, and the real effects may be greater than those found in this ITC. This uncertainty was at least partially addressed by SA5, where patients on RWT who changed their treatment during the study were counted as non-responders, and the resulting OR and RR were numerically higher than in the main analysis.

### 4.5. Clinical relevance and implications of findings

The focus of this study on the clinically meaningful outcomes of response and remission contributes towards the real-world relevance of these findings (1, 14, 41). Moreover, RWT in the EOTC is a clinically meaningful non-interventional comparator, with individualised treatments prescribed by clinicians representing what is expected to be the best available choice for each patient. Finally, the reporting of NNT, as recommended by the American College of Neuropsychopharmacology (42), helps to determine clinically significant differences between treatments and allows estimation of the relative gain in the use of esketamine NS compared with RWT. NNT values showed that for every five patients treated with esketamine NS and five patients treated with RWT, one additional patient on esketamine NS achieved response, and that for every seven patients treated with esketamine NS and the same number treated with RWT, one additional patient on esketamine NS achieved remission. Together, these features underline the clinical relevance of the findings reported here. Moreover, current RWT options result in a high likelihood of treatment failure for patients with TRD that increases as the number of treatment failures increases (8). Since TRD has substantial clinical and societal impact (8, 10–12), including high levels of unemployment and poor productivity and functionality (8), more effective alternatives will be of critical relevance in the future.

### 4.6. Future directions

The analysis presented here supports the long-term superiority of esketamine NS over RWT. Given the non-randomised nature of the comparisons reported, additional randomised studies will be important to further support the findings from ICEBERG. While ICEBERG suggests benefit of esketamine NS over a heterogeneous mixture of treatments, this improved response may not be observed when comparing against each different individual treatment. Gathering more evidence against a comparison group with a more homogenous pool of treatments will help determine if esketamine NS is still superior to specific treatment strategies. To this end we have carried out an additional analysis from ICEBERG, comparing esketamine NS to polypharmacy strategies (combination and augmentation therapies) from the EOTC (25). Furthermore, ESCAPE-TRD (NCT04338321), a study to compare esketamine NS with extended-release quetiapine currently ongoing (43), will provide comparative evidence with this commonly prescribed augmentation treatment.

This adjusted ITC of 6-month response and remission data provides strong evidence that esketamine NS has a significant and sustained benefit over RWT for patients with TRD. The robustness of the ITC is supported by similar baseline characteristics, as well as the fact that adjustment for multiple covariates, and extensive sensitivity and threshold analyses, did not change the conclusions of esketamine NS superiority over RWT. These results support that esketamine NS may be a more effective alternative for TRD than existing RWT, and thus contribute to address the substantial, long-term unmet needs of patients with this condition, with a wide-reaching impact on patients, caregivers and society as a whole.

## Animated summary

To view an animated summary of this publication, please click on the Supplementary video, or visit the manuscript online at: https://doi.org/10.3389/fpsyt.2023.1250980.

## Data availability statement

The raw data supporting the conclusions of this article will be made available by the authors, without undue reservation.

## Ethics statement

Ethical approval was not required for the studies involving humans because as this publication reports findings from an indirect treatment comparison, ethical approval was previously obtained for each individual study. Further details can be found in the respective primary publications. The studies were conducted in accordance with the local legislation and institutional requirements. Written informed consent for participation was not required from the participants or the participants’ legal guardians/next of kin in accordance with the national legislation and institutional requirements because as this publication reports findings from an indirect treatment comparison, written informed consent from patients was not required for this analysis.

## Author contributions

AJOM, JM, BR, YG, JC, NP, SB, and SMH: substantial contributions to study conception and design, substantial contributions to analysis and interpretation of the data and drafting the article or revising it critically for important intellectual content. All authors contributed to the article and approved the submitted version.
