# Supplementary material for: ICEBERG study: an indirect adjusted comparison estimating the long-term benefit of esketamine nasal spray when compared with routine treatment of treatment resistant depression in general psychiatry
Source: Front Psychiatry. 2023 Oct 31;14:1250980. doi: 10.3389/fpsyt.2023.1250980 (PMC10669153; doi:10.3389/fpsyt.2023.1250980)
Supplement: Supplementary file 2 [file Data_Sheet_1.DOCX]

Supplementary Material

ICEBERG Study: an Indirect Adjusted Comparison Estimating the Long-Term Benefit of Esketamine Nasal Spray when Compared with Routine Treatment of Treatment Resistant Depression in General Psychiatry

Oliveira-Maia AJ, Morrens J, Rive B,^*^ Godinov Y, Cabrieto J, Perualila N, Barbreau S, Mulhern Haughey S

*** Correspondence:** Benoit Rive: brive@its.jnj.com

# Supplementary Figures and Tables

## Supplementary Tables

### Supplementary Table 1. Summary of methodologies in the EOTC and SUSTAIN-2

|  | | **EOTC** | | **SUSTAIN-2** | |
| --- | --- | --- | --- | --- | --- |
| **Eligibility criteria** | | - ≥18 years - Diagnosis of MDD without psychotic features - Non-response to ≥2 antidepressants in the current episode - MADRS ≥22 at screening | | | |
| **Treatment strategy** | | Patients initiated a new, routine treatment for TRD. Monotherapy, combination therapy and augmentation therapy were all allowed, and patients could switch treatments and continue in the study. | | 56/84mg esketamine NS 2x weekly for four weeks, then weekly/every two weeks all alongside a newly initiated SSRI/SNRI | |
| **Follow-up (frequency, inpatient, outpatient)** | | Baseline data collection and a 12-month observational period with a minimum follow-up of approximately 6 months for each enrolled patient, plus an extended observation period up to 6 months from recruitment of the last patient | | Re-evaluation at four‑week intervals | |
| **Relevant outcome** | | Change in MADRS total score | | | |

IPTW: inverse probability of treatment weighting; MADRS: Montgomery-Åsberg Depression Rating Scale; MDD:‍ major depressive disorder; NS:‍ nasal spray; SNRI: serotonin‑norepinephrine reuptake inhibitor; SSRI:‍ selective serotonin reuptake inhibitor; TRD: treatment resistant depression.

### Supplementary Table 2. Covariates, ranked by expert medical opinion, used for PS IPW and logistic regression model adjustment comparison

| Rank | Covariate | **Categorisation** | **Notes** |
| --- | --- | --- | --- |
| 1 | Total number of failures in  current MDE | 2/3/≥4 | Based on MGH-ATRQ |
| 2 | Age, years | <30/30–44/45–64/≥65 | Data cut-off for ‘elderly patients’ at ≥65, other cut-offs determined by exploratory analysis |
| 3 | MADRS score, baseline | <31/31–34/>34 | Data cut-off >34 to identify patients with severe TRD (Müller et al., 2003), cut-off of <31 determined by exploratory analysis |
| 4 | Total number of MDE | 1/2–5/6–10/>10 | Based on medical history |
| 5 | Duration of current MDE, weeks | ≤32/33–51/52–103/≥104 | Based on medical history; data cut-offs of 33 and 52 weeks correspond to quartiles of distribution, cut-off of 104 weeks based on SUSTAIN-2 study protocol which stated ‘if single episode MDD, the duration must be ≥2 years’ |
| 6 | Gender | Male/Female |  |
| 7 | Prior failure on augmentation | No/Yes | Based on MGH-ATRQ |
| 8 | History of suicidality | No event/Suicidal ideation/Suicidal behaviour/Missing | Based on C-SSRS (lifetime) |
| 9 | Time since first diagnosis of MDD, years | <5/5–19/≥20 | Based on medical history; data cut-offs determined by exploratory analysis |
| Rank | Covariate | Categorisation | Notes |
| 10 | Age at diagnosis of MDD, years | <35/35–54/≥55 | Based on medical history; data cut-off of 55 corresponds to previously conducted post-hoc analysis of ‘late onset’ patients from TRANSFORM-3, cut-off of 35 determined by exploratory analysis |
| 11 | Prior failure on SSRI | No/yes | Based on MGH-ATRQ |
| 12 | Prior failure on SNRI |  |  |
| 13 | Prior failure on TCA |  |  |
| 14 | Prior failure on other AD^a^ |  |  |
| 15 | Average duration of each treatment line during current MDE, weeks | <12/12–23/24–51/≥52 | Every patient received multiple treatment lines during their current MDE, these data represent average duration of each individual treatment line and not overall duration of current MDE, data were calculated as duration of current MDE (based on medical history) divided by total number of failures in current MDE (based on MGH-ATRQ); data cut-offs correspond to quartiles of distribution |
| 16 | CGI-S score, baseline | 1–4/5/6–7/Missing |  |
| 17 | EQ-VAS score, baseline | <30/≥30/Missing |  |

All scored covariates measured at baseline. ^a^Prior failure on other AD included trazodone, nefazodone, vilazodone, bupropion, mirtazapine, mianserin, opipramol, agomelatine, tianeptine, reboxetine and vortioxetine. AD: antidepressant; CGI-S: Clinical Global Impression-Severity; C-SSRS: Columbia-Suicide Severity Rating Scale; EQ-VAS: EuroQoL-visual analogue scale; IPW: inverse probability weighting; MADRS: Montgomery-Åsberg Depression Rating Scale; MDD: major depressive disorder; MDE: major depressive episode; MGH-ATRQ: Massachusetts General Hospital Antidepressant Treatment Response Questionnaire; PS: propensity score; SNRI: serotonin-norepinephrine reuptake inhibitor; SSRI: selective serotonin reuptake inhibitor; TCA: tricyclic antidepressant; TRD: treatment resistant depression.

### Supplementary Table 3. Chance of 6-month response, main analysis and SAs 1–3

| ITC method | **Probability** | | **OR** | **RR** | **RD** | **NNT** |
| --- | --- | --- | --- | --- | --- | --- |
|  | **% (95% CI)** | | **(95% CI)** | **(95% CI)** | **(95% CI)** | **(95% CI)** |
|  | **Esketamine NS** | **RWT** | **p value** | **p value** | **p value** |  |
| Main analysis |  |  |  |  |  |  |
| ATT | 49.7 (45.6–53.9) | 26.4  (21.5–31.4) | 2.756  (2.034–3.733)  <0.0001 | 1.882  (1.534–2.310)  <0.0001 | 0.233  (0.169–0.298)  <0.0001 | 5  (4–6) |
| Alternative IPW adjustments | | | | | | |
| SA1 (ATC) | 43.1  (39.0–47.2) | 25.4  (20.5–30.3) | 2.224  (1.636–3.024)  <0.0001 | 1.696  (1.369–2.102)  <0.0001 | 0.177  (0.113–0.241)  <0.0001 | 6  (5–9) |
| SA2 (sATE) | 47.7  (43.4–52.1) | 26.0  (21.1–31.0) | 2.600  (1.908–3.542)  <0.0001 | 1.836  (1.488–2.266)  <0.0001 | 0.217  (0.152–0.283)  <0.0001 | 5  (4–7) |
| SA3 (ATO) | 46.8  (38.6–55.0) | 27.3  (20.0–34.7) | 2.338  (1.426–3.834)  0.0008 | 1.712  (1.243–2.358)  0.0010 | 0.195  (0.085–0.305)  0.0005 | 6  (4–12) |

Prior to IPW adjustments, 278/559 (49.7%) patients in the esketamine NS group and 78/307 (25.4%) patients in the RWT group experienced response at Month 6. Missing data were handled as per **Table 1**. Data in SA1 were adjusted using the ATC covariate adjustment method. Data in SA2 were adjusted using the sATE adjustment method. Data in SA3 were adjusted using the ATO adjustment method. All data were calculated using a non-linear mixed model, except SA3 which was calculated using usual formulas applied to reweighted data. ATC: average treatment effect among control; ATO: average treatment effect among the overlap population; ATT: rescaled average treatment effect among treated; CI: confidence interval; IPW: inverse probability weighting; ITC:‍ indirect treatment comparison; NNT: number needed to treat; NS: nasal spray; OR: odds ratio; RD: risk difference; RR: relative risk; RWT: real-world treatment; SA: sensitivity analysis; sATE: stabilised average treatment effect.

### Supplementary Table 4. Chance of 6-month remission, main analysis and SAs 1–3

| ITC method | **Probability** | | | | | | **OR** | | **RR** | | **RD** | | **NNT** |
| --- | --- | --- | --- | --- | --- | --- | --- | --- | --- | --- | --- | --- | --- |
|  | **% (95% CI)** | | | | | | **(95% CI)** | | **(95% CI)** | | **(95% CI)** | | **(95% CI)** |
|  | **Esketamine NS** | | | | **RWT** | | **p value** | | **p value** | | **p value** | |  |
| Main analysis | |  | |  | |  | |  | |  | |  | |
| ATT | | 33.6  (29.7–37.6) | | 18.2  (13.9–22.5) | | 2.276  (1.621–3.196)  <0.0001 | | 1.847  (1.418–2.406)  <0.0001 | | 0.154  (0.096–0.213)  <0.0001 | | 7  (5–11) | |
| Alternative IPW adjustments | | |  | | |  | |  | |  | |  | |
| SA1 (ATC) | | 29.4  (25.6–33.2) | | 16.3  (12.1–20.4) | | 2.140  (1.502–3.050)  <0.0001 | | 1.805  (1.358–2.400) <0.0001 | | 0.131  (0.075–0.187)  <0.0001 | | 8  (6–14) | |
| SA2 (sATE) | | 32.4  (28.3–36.4) | | 17.5  (13.3–21.8) | | 2.251  (1.588–3.190)  <0.0001 | | 1.846  (1.403–2.428) <0.0001 | | 0.148  (0.090–0.207)  <0.0001 | | 7  (5–12) | |
| SA3 (ATO) | | 31.8  (24.1–39.5) | | 17.1  (10.9–23.3) | | 2.261  (1.289–3.965)  0.0044 | | 1.860  (1.204–2.873)  0.0052 | | 0.147  (0.049–0.245)  0.0034 | | 7  (5–21) | |

Prior to IPW adjustments, 186/559 (33.6%) patients in the esketamine NS group and 50/307 (16.3%) patients in the RWT group experienced remission at Month 6. Missing data were handled as per **Table 1**. Data in SA1 were adjusted using the ATC covariate adjustment method. Data in SA2 were adjusted using the sATE adjustment method. Data in SA3 were adjusted using the ATO adjustment method. All data were calculated using a non-linear mixed model, except SA3 which was calculated using usual formulas applied to reweighted data. ATC: average treatment effect among control; ATO: average treatment effect among the overlap population; ATT: rescaled average treatment effect among treated; CI: confidence interval; IPW: inverse propensity weighting; ITC: indirect treatment comparison; NNT: number needed to treat; NS: nasal spray; OR: odds ratio; RD: risk difference; RR: relative risk; RWT: real-world treatment; SA: sensitivity analysis; sATE: stabilised average treatment effect.

### Supplementary Table 5. Chance of 6-month response and remission, sensitivity analyses (SA) 4, 5 and 6

| Sensitivity analysis | **Outcome** | **Probability** | | **OR** | **RR** | **RD** | **NNT** | |
| --- | --- | --- | --- | --- | --- | --- | --- | --- |
|  |  | **% (95% CI)** | | **(95% CI)** | **(95% CI)** | **(95% CI)** | **(95% CI)** | |
|  |  | **Esketamine NS** | **RWT** | **p value** | **p value** | **p value** | **p value** | |
| **SA4^a^** | | | | | | | |  |
|  | **Response** | 43.7 (40.0–47.4) | 25.5 (20.6–30.3) | 2.270 (1.685–3.058) <0.0001 | 1.715 (1.391–2.115) <0.0001 | 0.182 (0.121–0.243) <0.0001 | 6 (5–9) | |
|  | **Remission** | 29.0 (25.6–32.4) | 17.3 (13.1–21.5) | 1.954 (1.393–2.743)  0.0001 | 1.677 (1.279–2.200)  0.0002 | 0.117 (0.063–0.172)  <0.0001 | 9 (6–16) | |
| **SA5^b^** | | | | | | | |  |
|  | **Response** | 49.7 (45.6–53.9) | 17.2 (13.0–21.4) | 4.754 (3.392–6.662) <0.0001 | 2.887 (2.232–3.734) <0.0001 | 0.325 (0.266–0.384) <0.0001 | 4 (3–4) | |
|  | **Remission** | 33.6 (29.7–37.6) | 14.3 (10.4–18.2) | 3.037 (2.114–4.364) <0.0001 | 2.352 (1.750–3.161) <0.0001 | 0.193 (0.138–0.249) <0.0001 | 6 (5–8) | |
| **SA6^c^** | | | | | | | |  |
|  | **Response** | 49.7 (45.6–53.9) | 28.6 (23.8–33.5) | 2.466 (1.847–3.294) <0.0001 | 1.737 (1.439–2.098) <0.0001 | 0.211 (0.147–0.275) <0.0001 | 5 (4–7) | |
|  | **Remission** | 33.6 (29.7–37.6) | 19.4 (15.1–23.6) | 2.108 (1.526–2.912) <0.0001 | 1.735 (1.355–2.223) <0.0001 | 0.143 (0.085–0.200) <0.0001 | 8 (5–12) | |

Prior to IPW adjustments, response and remission at Month 6 were: ^a^SA4: 301/689 (43.7%) and 200/689 (29.0%) for patients in the esketamine group and 78/307 (25.4%) and 53/307 (17.3%) for patients in the RWT group; ^b^SA5: 278/559 (49.7%) and 188/559 (33.6%) for patients in the esketamine group and 54/313 (17.2%) and 45/313 (14.3%) for patients in the RWT group; ^c^SA6: 278/559 (49.7%) and 188/559 (33.6%) for patients in the esketamine group and 96/336 (28.6%) and 65/336 (19.4%) for patients in the RWT group. Main analysis ITC method (ATT) only. Missing data were handled as per Table 1. ATT: rescaled average treatment effect among treated; CI: confidence interval; IPW: inverse propensity weighting; ITC:‍ indirect treatment comparison; NNT: number needed to treat; OR: odds ratio; NS: nasal spray**;** RD: risk difference; RR: relative risk; RWT: real-world treatment; SA: sensitivity analysis.

### Supplementary Table 6. Threshold analysis based on OR, RR and RD for chance of 6-month response and remission

| Outcome | Efficacy measure | **Probability,  % (95% CI)** | | **Difference,^b^ %** |
| --- | --- | --- | --- | --- |
|  |  | **Observed** | **Lowest simulated significant result^a^** |  |
| Remission |  |  |  |  |
|  | OR | 33.6 (29.7–37.6) | 24.1 (20.6–27.7) | 9.5 |
|  | RD | 33.6 (29.7–37.6) | 24.0 (20.4–27.5) | 9.7 |
|  | RR | 33.6 (29.7–37.6) | 24.1 (20.6–27.7) | 9.5 |
| **Response** |  |  |  |  |
|  | OR | 49.7 (45.6–53.9) | 32.9 (29.0–36.8) | 16.8 |
|  | RD | 49.7 (45.6–53.9) | 32.7 (28.8–36.6) | 17.0 |
|  | RR | 49.7 (45.6–53.9) | 33.1 (29.2–37.0) | 16.6 |

Missing data were handled as per **Table 1**. ^a^Pre-determined significance value was p<0.05. ^b^Maximum difference in response/remission before loss of significance in outcomes. CI: confidence interval; OR: odds ratio; RD: risk difference; RR: relative risk.

## Supplementary Figures

### Supplementary Figure 1. Study diagrams for EOTC and SUSTAIN-2

1. SUSTAIN-2 study design

**
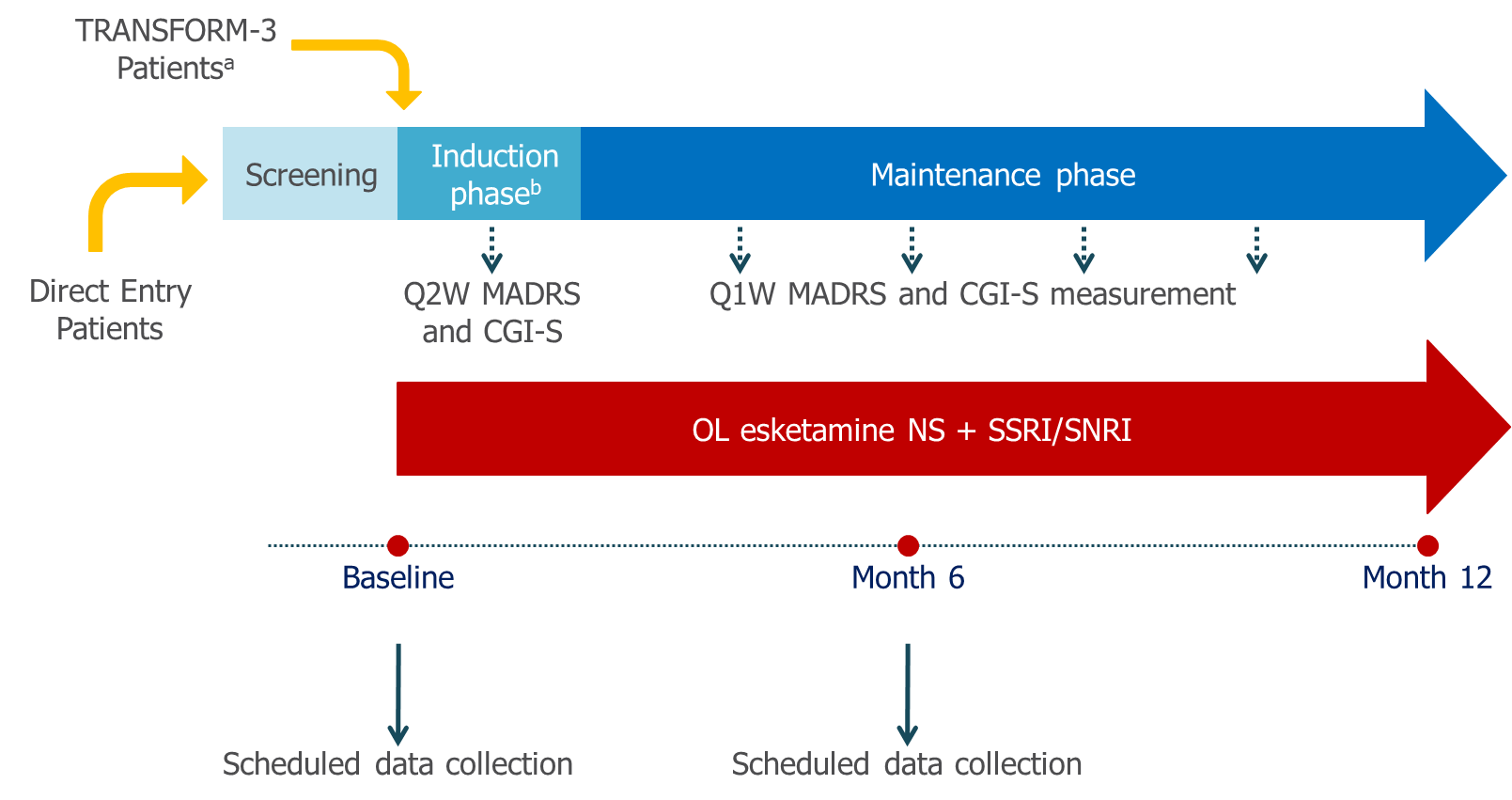
**

1. EOTC study design


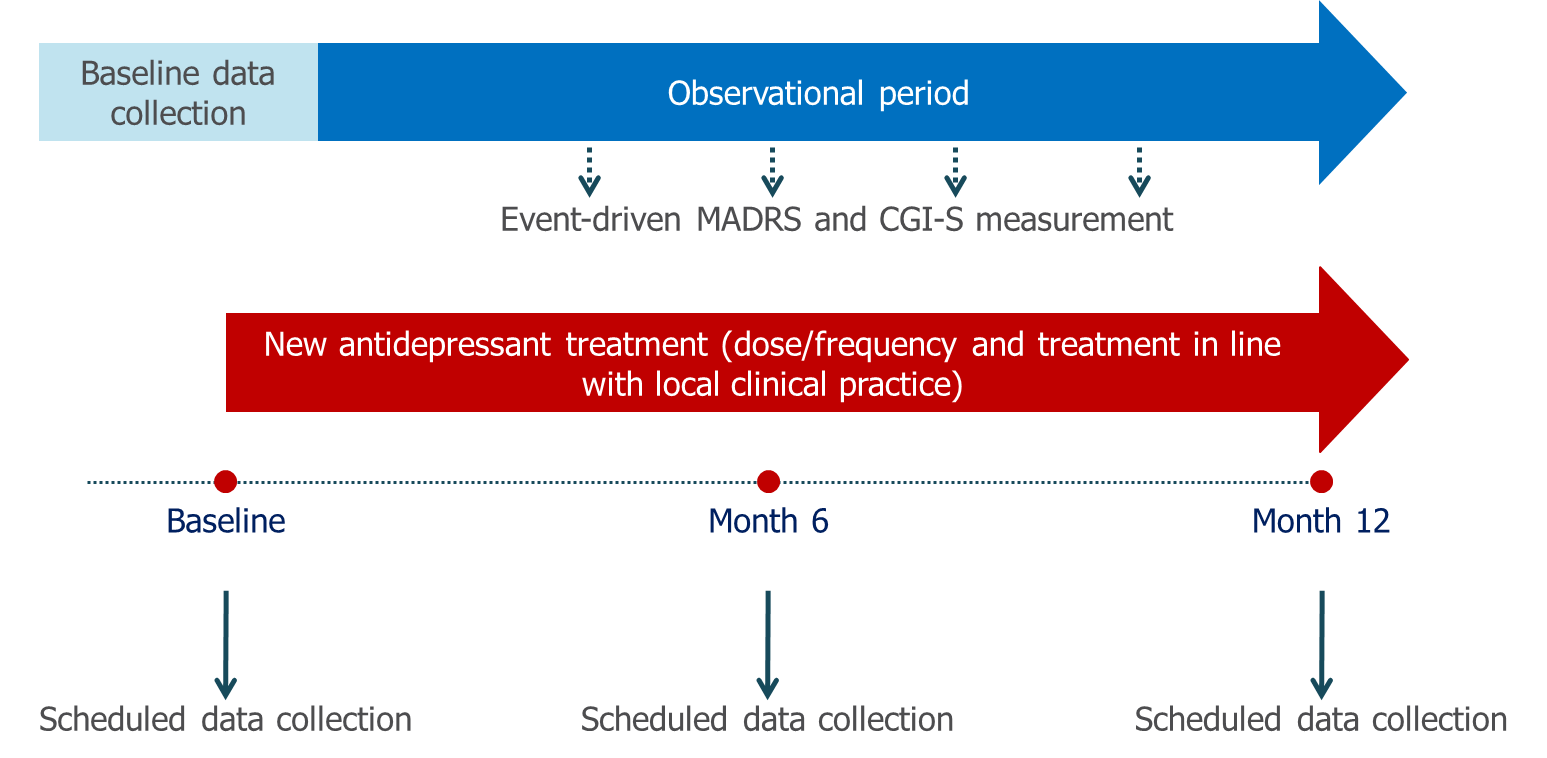


SUSTAIN-2 study design (**A**) and EOTC study design (**B**). ^a^TRANSFORM-3 patients in the SUSTAIN-2 study were excluded from the ICEBERG analysis, as those in TRANFORM-3 were continuing a treatment and not direct entry. ^b^Only responders continued to the maintenance phase. The EOTC study was terminated when the last enrolled patient reached Month 6; SUSTAIN-2 was terminated when ≥300 and ≥100 patients had received esketamine NS for 6 and 12 months, respectively. When this point was reached, all patients still in SUSTAIN-2 were withdrawn, with ‘study terminated by sponsor’ cited as reason for withdrawal. CGI-S: Clinical Global Impression-Severity; EOTC: European Observational TRD Cohort; MADRS: Montgomery-Åsberg Depression Rating Scale; NS: nasal spray; OL: open-label; SNRI: serotonin‑norepinephrine reuptake inhibitor; SSRI: selective serotonin reuptake inhibitor; TRD: treatment resistant depression.

### Supplementary Figure 2. Study flow diagrams for patients included in ICEBERG from SUSTAIN-2 and EOTC

1. SUSTAIN-2 (esketamine NS)

**
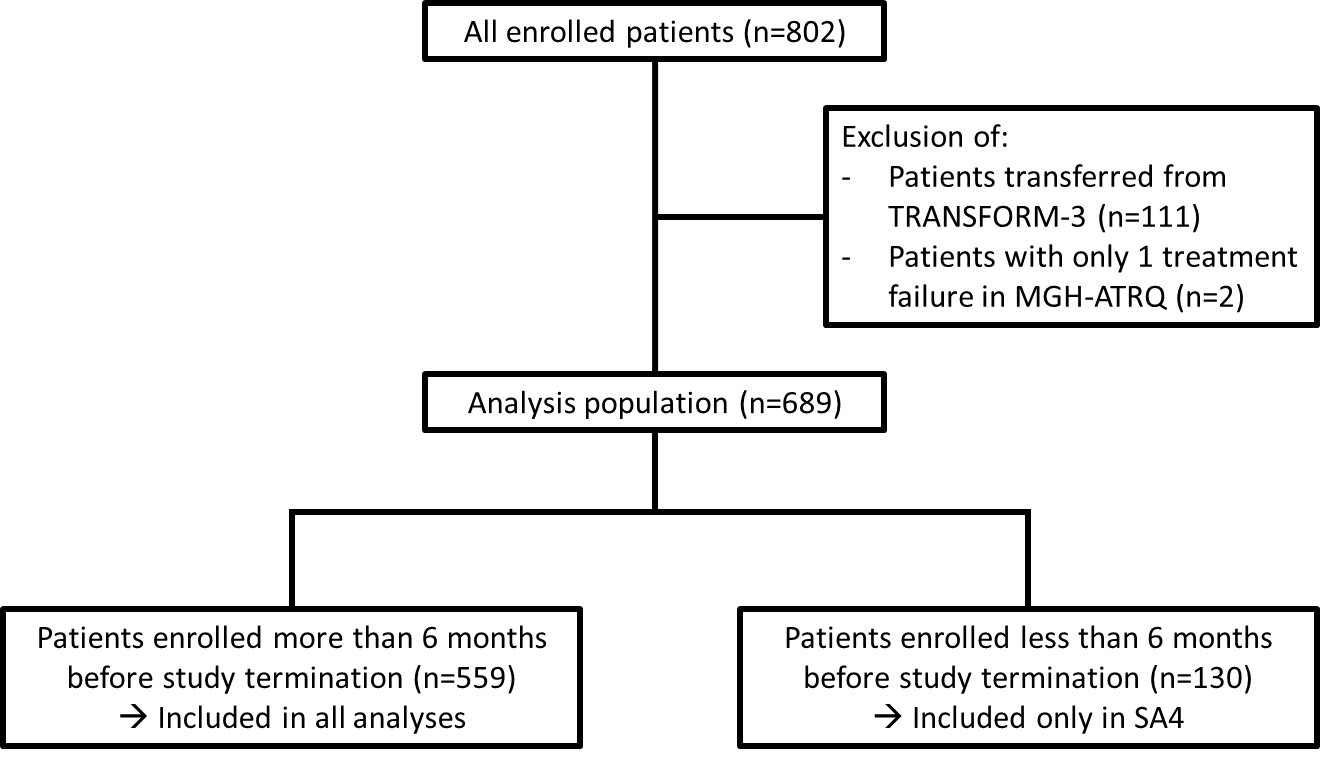
**

1. EOTC (RWT)


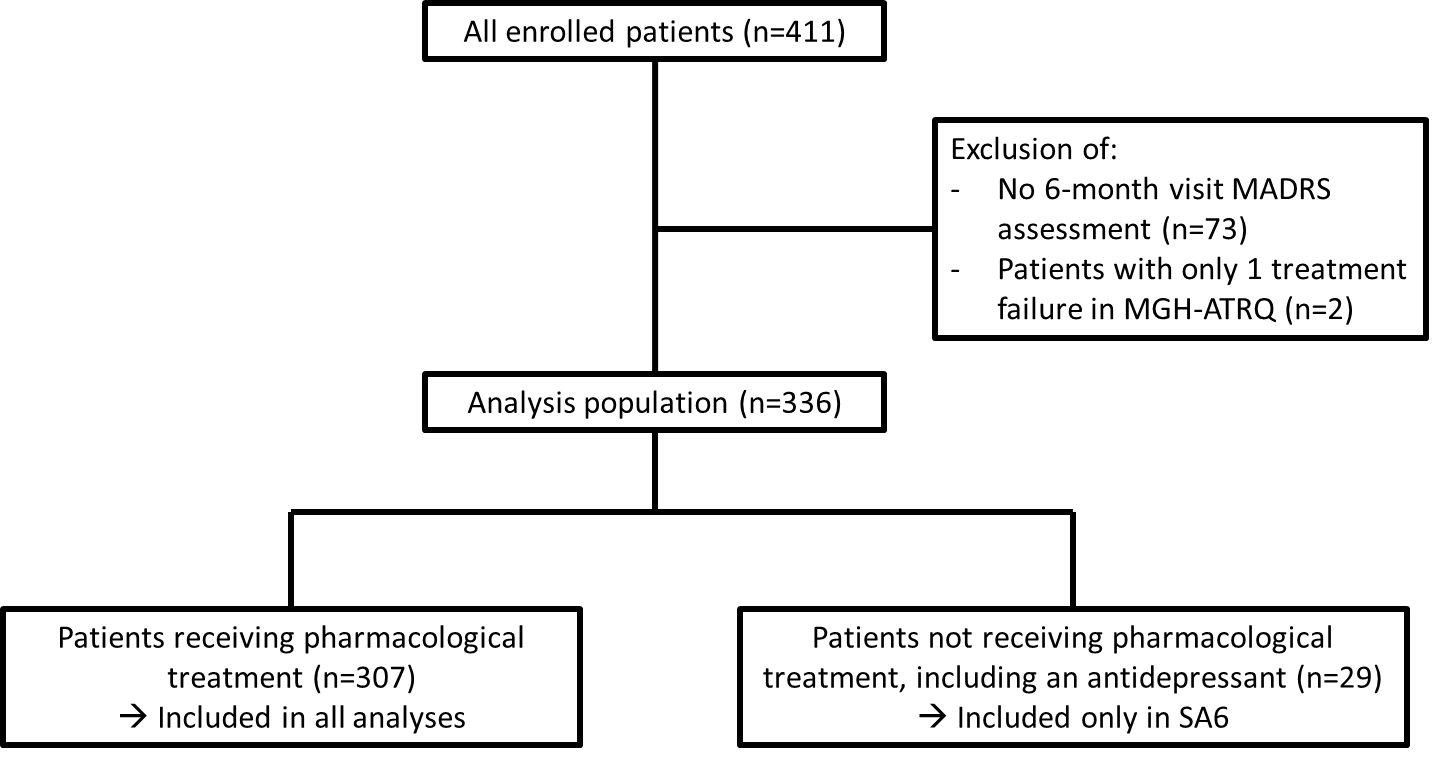


Patient disposition in SUSTAIN-2 (**A**) and EOTC (**B**). EOTC: European Observational TRD Cohort; MADRS: Montgomery‑Åsberg Depression Rating Scale; MGH-ATRQ: Massachusetts General Hospital Antidepressant Treatment Response Questionnaire; NS: nasal spray; RWT: real world treatment; SA: sensitivity analysis; TRD: treatment resistant depression.

### Supplementary Figure 3. Distribution of propensity scores by treatment

1. Before reweighting


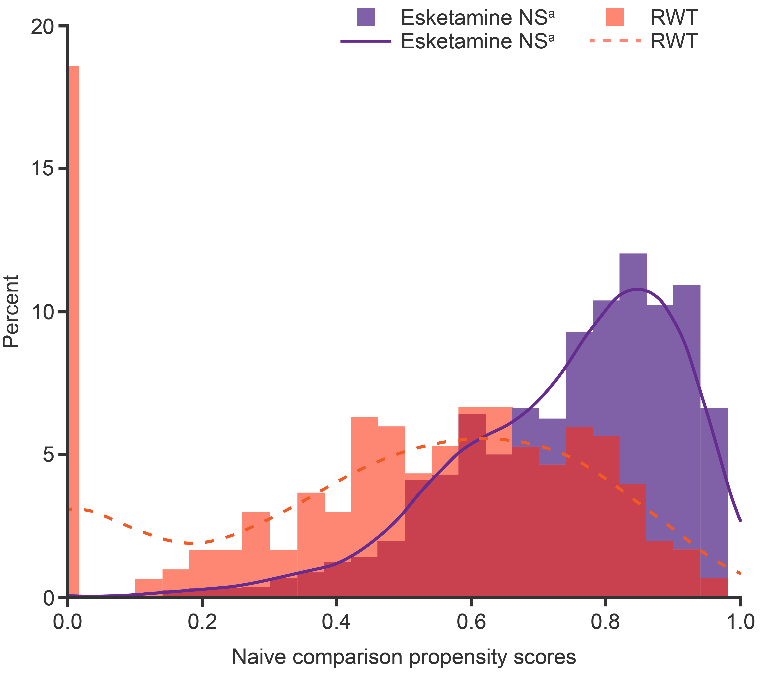


1. After reweighting


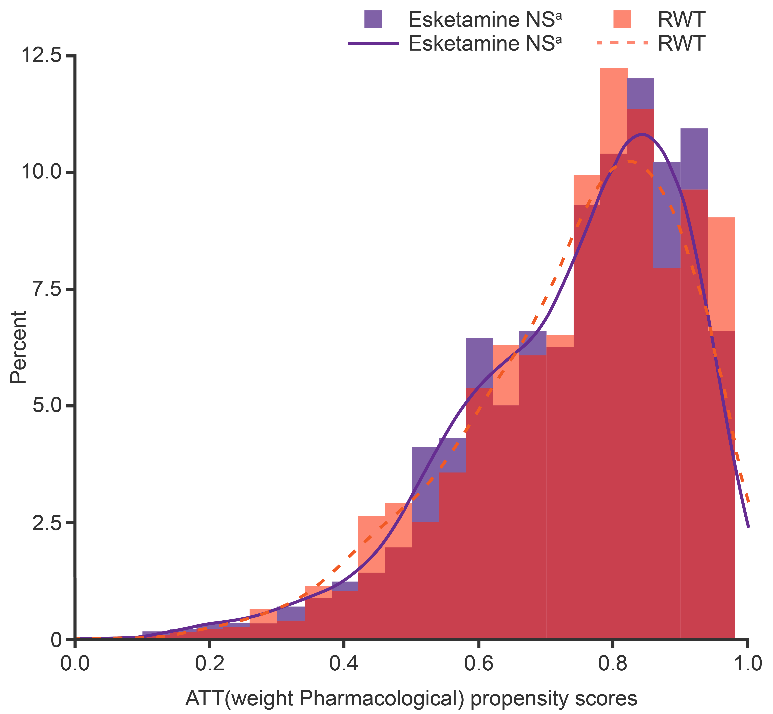


Naïve (**A**) and reweighted (**B**) distributions of propensity scores. ^a^Esketamine NS in addition to an SSRI/SNRI. ATT: rescaled average treatment effect among treated; NS: nasal spray; RWT: real‑world treatment; SNRI: serotonin-norepinephrine reuptake inhibitor; SSRI: selective serotonin reuptake inhibitor.

### References

Müller, M.J., Himmerich, H., Kienzle, B., Szegedi, A., 2003. Differentiating moderate and severe depression using the Montgomery-Asberg depression rating scale (MADRS). J. Affect. Disord. 77(3), 255-260. https//doi:10.1016/s0165-0327(02)00120-9

# Supplementary Video Abstract

<<Ask editors to embed animation here>>
